# Role of a circadian-relevant gene NR1D1 in brain development: possible involvement in the pathophysiology of autism spectrum disorders

**DOI:** 10.1038/srep43945

**Published:** 2017-03-06

**Authors:** Masahide Goto, Makoto Mizuno, Ayumi Matsumoto, Zhiliang Yang, Eriko F. Jimbo, Hidenori Tabata, Takanori Yamagata, Koh-ichi Nagata

**Affiliations:** 1 Department of Pediatrics, Jichi medical university, Tochigi, Japan; 2 Department of Molecular Neurobiology, Institute for Developmental Research, Aichi Human Service Center, Kasugai, Japan; 3 Department of Neurochemistry, Nagoya University Graduate School of Medicine, Nagoya, Japan

## Abstract

In our previous study, we screened autism spectrum disorder (ASD) patients with and without sleep disorders for mutations in the coding regions of circadian-relevant genes, and detected mutations in several clock genes including *NR1D1*. Here, we further screened ASD patients for *NR1D1* mutations and identified three novel mutations including a *de novo* heterozygous one c.1499 G > A (p.R500H). We then analyzed the role of Nr1d1 in the development of the cerebral cortex in mice. Acute knockdown of mouse Nr1d1 with *in utero* electroporation caused abnormal positioning of cortical neurons during corticogenesis. This aberrant phenotype was rescued by wild type Nr1d1, but not by the c.1499 G > A mutant. Time-lapse imaging revealed characteristic abnormal migration phenotypes in Nr1d1-deficient cortical neurons. When Nr1d1 was knocked down, axon extension and dendritic arbor formation of cortical neurons were also suppressed while proliferation of neuronal progenitors and stem cells at the ventricular zone was not affected. Taken together, Nr1d1 was found to play a pivotal role in corticogenesis via regulation of excitatory neuron migration and synaptic network formation. These results suggest that functional defects in NR1D1 may be related to ASD etiology and pathophysiology.

Autism spectrum disorder (ASD) is a developmental disorder characterized by varying degrees of deficits in social interaction, and restricted and repetitive behaviors^1^. It is currently estimated that 1.5% of children are diagnosed with ASD^2^. ASD is associated with problems in the early developmental period and frequently accompanied by comorbid conditions, including hyperactivity, panic, self-injury and sleep disturbance. Among them, sleep disruption, such as insomnia or a short sleep cycle, is one of the most common and distressful problems^3^,^4^. A study, based on parental reports, estimated that 44–83% of children with ASD^5^ have sleep problems, compared with 25–40% of typically developing children^6^.

The circadian rhythm is a fundamental regulatory factor in cells throughout the bodies of most organisms^7^. In mammals, the autonomous rhythm of the individual cell is entrained by hormonal and neuronal signals from a central circadian clock located in the suprachiasmatic nuclei of the hypothalamus, and is reset daily by light^8^. The core circadian clock mechanism is composed of two interlocked transcriptional negative feedback loops^9^. In the primary loop, transcriptional activators, Bmal1 (Arntl) and Clock (or its ortholog Npas2), form a DNA-binding heterodimer and drive expression of Per1/2/3 and Cry1/2 genes. These protein products ultimately feed back to repress Bmal1-Clock activity. This loop also drives rhythmic expression of the nuclear hormone receptors, Nr1d1 (nuclear receptor subfamily 1 group D member 1, also known as Rev-Erbα) and Nr1d2 (Rev-Erbβ), which in turn rhythmically repress the expression of Bmal1 and Clock as the second loop^10^,^11^. Recently, abnormalities of circadian-relevant genes such as *PER1, CLOCK, MTNR1A* and *MTNR1B* have been reported to be associated with the pathogenecity of ASD^12^,^13^,^14^. We also recently demonstrated that mutations in *NR1D1* and other various circadian-relevant genes possibly affecting their product functions were more frequent in ASD patients than in matching controls^15^. Thus, circadian-relevant proteins were likely to be involved in brain development, and the impaired molecular clock mechanisms may possibly contribute to the etiology of ASD.

*NR1D1* is located on 17q11.2, which was shown to be a ASD susceptibility region^16^,^17^ and has been demonstrated to regulate the transcription of target genes^18^,^19^. As for neuronal function, while synaptic activity induced the distribution of Nr1d1 to the spine and dendrites in wild-type mice^20^, Nr1d1*-*knockout mice displayed abnormal behaviors such as marked hyperactivity, impaired response habituation in novel environments, deficient contextual memories and impairment in nest-building ability^21^. The above observations raise the possibility that NR1D1 is crucial for synaptic functions and is a causal gene candidate for ASD and other neurodevelopmental disorders.

In the present study, we identified three novel mutations in *NR1D1* in ASD patients, and performed *in vivo* analyses to elucidate the role of Nr1d1 in corticogenesis. While Nr1d1 did not appear to participate in neuronal cell proliferation, its deficiency was shown to impair migration, axon growth and dendritic arbor development of excitatory pyramidal neurons. These results strongly suggest a role of NR1D1 in brain development and its involvement in the etiology of ASD.

## Results

### Sequence analyses

**Figure 1:**
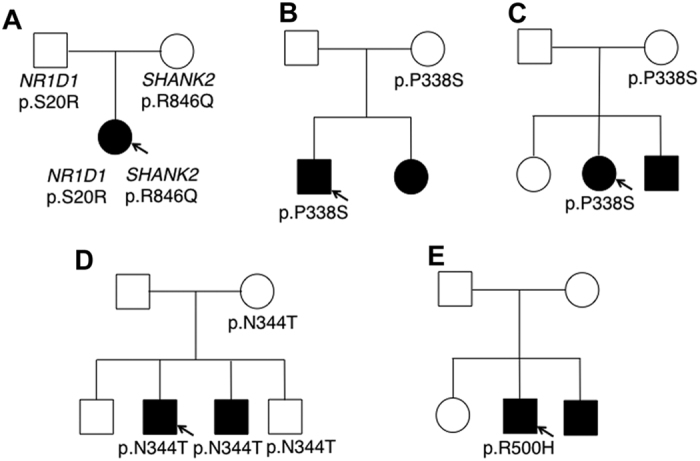
Pedigree analyses. (**A**) The p.S20R mutation in *NR1D*1 and p.R846Q mutation in *SHANK2* was detected in the patient. Each mutation was inherited from separate unaffected parents. (**B–D**) Mutations were inherited from their unaffected mothers. (**E**) The p.R500H mutation was determined to be *de novo*. The mutation was not detected in the younger brother with ASD.

**Figure 2:**
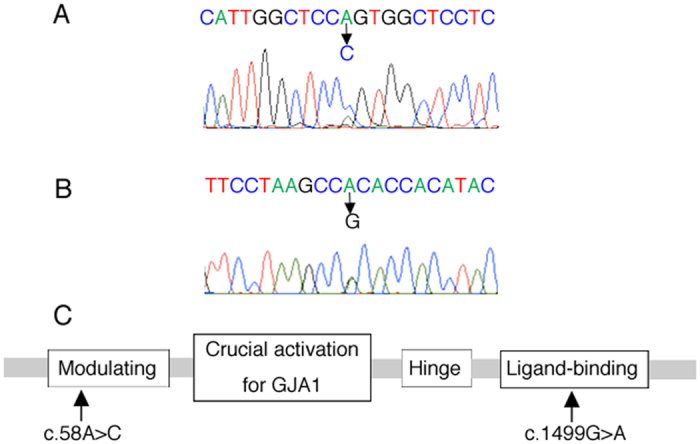
Sequence analyses of the patients with the c.58A > C and c.1499G > A mutation. **(A,B)** Genomic DNA sequence chromatograms. Positions of the c.58A > C **(A)** and c.1499G > A **(B)** mutations are indicated. **(C)** Schematic representation of the structure of Nr1d1. c.58A > C and c.1499G > A mutations were detected in the Modulating and Ligand-binding domain, respectively. GJA1, Gap junction alpha-1 protein.

By direct sequencing analyses of ASD patients, we detected single base changes with an amino acid substitution in the coding region of *NR1D1* in four individuals (Table 1). These mutations were not detected in the Japanese and Caucasian controls. Among the mutations detected, c.1012C > T (p.P338S) and c.1031A > C (p.N344T) were identified as the rare SNPs rs143682026 (A = 0.0040/20) and rs145435357 (G = 0.0008/4), respectively. Variant effect prediction tools found the c.1012C > T mutation to most likely be non-pathogenic and only one tool suggested c.1031A > C to be disease causing (Table 1). In addition, neither mutation was related to the disease in the pedigree analyses (Fig. 1). On the other hand, we recently reported on a patient with another missense mutation, c.58A > C (p. S20R) (Figs 1A and 2A)^15^, and this mutation was also detected in the father. The patient also presented an additional missense c.2537G > A mutation (p.R846Q) in *SHANK2* inherited from her mother which encodes a synaptic scaffold protein. The *NR1D1* c.58A > C mutation could impair gene product function by Mutation Tester analysis. The father also carried the mutation c.1673C > T (p.S558L), rs201903415 (A = 0.00002/2). This mutation was not detected in the patient, but was detected in 3 out of 121 samples in the Japanese controls. Meanwhile, we identified a novel *de novo* heterozygous missense mutation, c.1499G > A, causing an arginine to histidine substitution (p.R500H) in one Caucasian sample (Figs 1E and 2B). This mutation was predicted to disrupt NR1D1 protein function by all three pathogenic analyses (Table 1). Interestingly, the c.1499G > A mutation is located in the ligand-binding region of NR1D1 (UniProt; http://www.uniprot.org/uniprot/P20393). Other mutations in *NR1D1* were not detected in the families analyzed in this study.

As for clinical symptoms, the patient with the c.1499G > A (p.R500H) mutation (AU1098302) had typical features of ASD. He could not maintain eye contact and appreciate emotions of others. He could speak unilaterally but could not communicate with others. He had repetitive and stereotypical speech, but no ritualistic movements. His memory was very well developed. Despite the mutation in a clock gene, he had no difficulty in sleep induction. Although the patient had an ASD-affected brother, the c.1499G > A mutation was not detected in the brother and his phenotype was a little bit different. The brother had strong anxiety and little sociability without verbal communication with others. Based on the genetic results and clinical features of the patient, we focused on the effects of NR1D1 deficiency on cortical development and analyzed the pathophysiological significance of the c.1499G > A mutation.

### Characterization of RNAi vectors and expression plasmids

**Figure 3:**
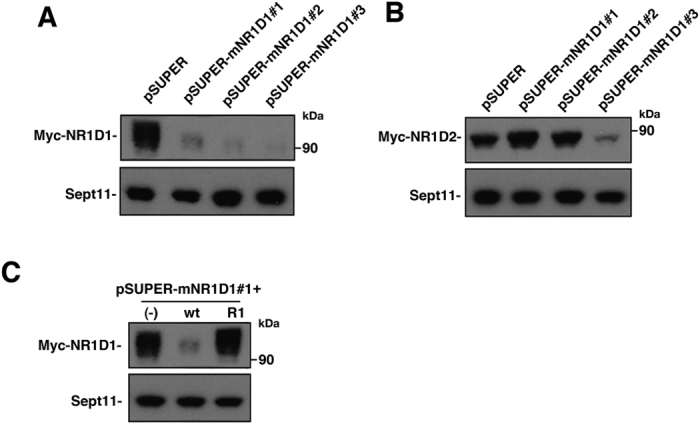
Characterization of RNAi vectors for Nr1d1. (**A**) pCAG-Myc-Nr1d1 or (**B**) pCAG-Myc-Nr1d2 was co-transfected into COS7 cells with control pSUPER vector, pSUPER-mNR1D1#1 - #3. After 48 h, cells were harvested and subjected to western blotting (20 μg protein per lane) with anti-Myc. Anti-Sept11 was used for a loading control. (**C**) pCAG-Myc vector (−), pCAG-Myc-Nr1d1 (wt) or –Nr1d1-R (R) was co-transfected into COS7 cells with control pSUPER vector (−) or pSUPER-mNR1D1#1 (wt, R). Analyses were done as in (**A)**.

From the genetic analyses of the patient, *NR1D1* could possibly be a contributing gene for the ASD phenotype. To test this possibility with *in vivo* analyses, we designed three RNAi vectors, pSUPER-mNR1D1#1, #2, and #3, against distinct regions in the mouse *Nr1d1* coding sequence. All the three vectors efficiently knocked down Nr1d1 expression in COS7 cells (Fig. 3A). While pSUPER-mNR1D1#1 and #2 did not affect the expression of the Nr1d1-related molecule, Nr1d2, pSUPER-mNR1D1#3 knocked down Nr1d2 as well (Fig. 3B). We prepared an RNAi-resistant version of Nr1d1, Nr1d1-R, and confirmed its resistance to pSUPER–mNR1D1#1 (Fig. 3C). We used Nr1d1-R in the rescue experiments.

### Expression of Nr1d1 in the developing mouse brain

**Figure 4:**
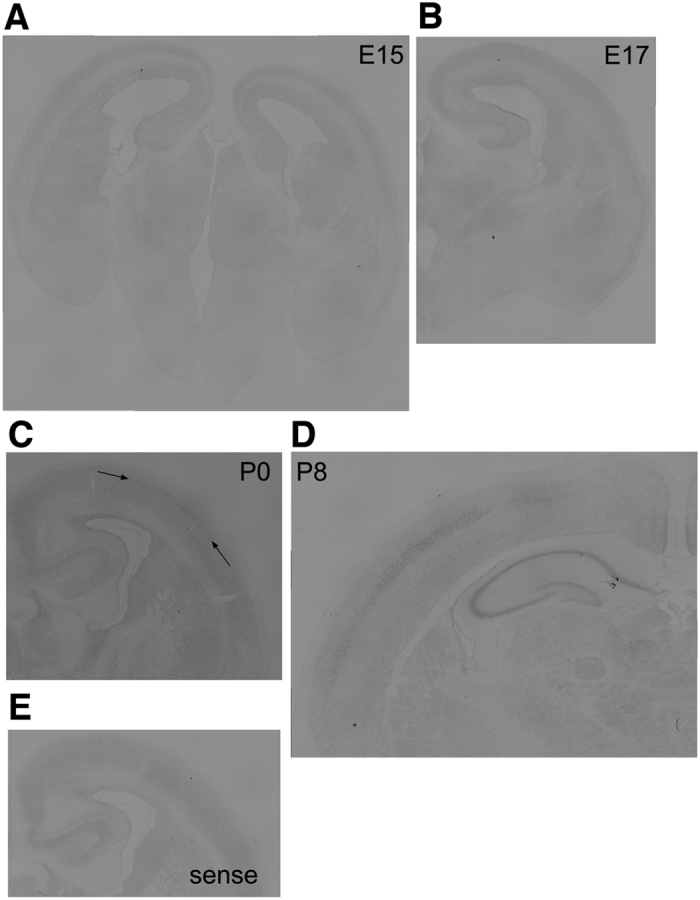
*In situ* hybridization of Nr1d1 in developing mouse brain. Coronal sections were examined for Nr1d1-mRNA at E15 **(A)**, E17 **(B)**, P0 **(C)** and P8 **(D)**. Layer IV was shown in **(C)** by *arrowheads.* **(E)** Sense control cRNA probe was used for P0 sample. Bars, 200 μm.

We first examined mRNA expression profiles of Nr1d1 through *in situ* hybridization during mouse brain development. Nr1d1 showed considerably low expression in the subventricular zone (SVZ)/ventricular zone (VZ) and cortical plate (CP) from E15 to P8 (Fig. 4A–D). It is notable that Nr1d1 appeared to be expressed moderately in layer IV of the somatosensory area at P0 and P8 (Fig. 4C *arrowheads* and D) when compared to the control experiment (Fig. 4E). At P8, relatively strong expression was detected in the hippocampus (Fig. 4D).

### Role of Nr1d1 in excitatory neuron migration during corticogenesis

**Figure 5:**
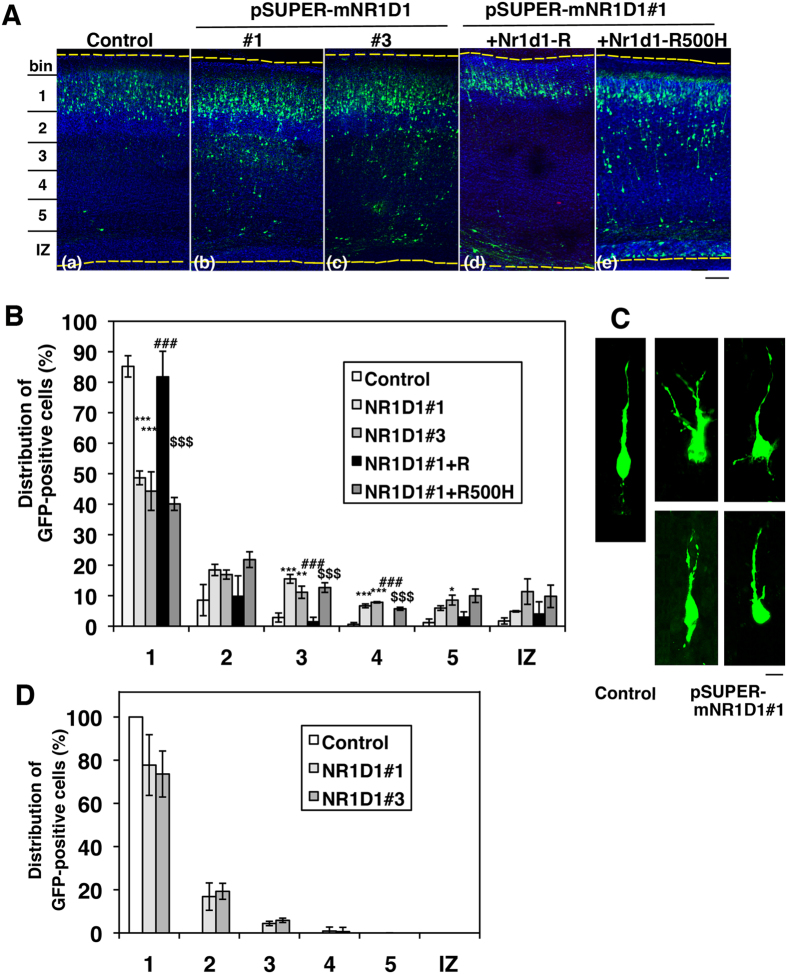
Effects of Nr1d1-knockdown on neuronal migration during corticogenesis. **(A)** pCAG-GFP was coelectroporated with control pSUPER vector (a), pSUPER-mNR1D1 #1 (b) or #3 (c) into cerebral cortices at E14.5. For the rescue experiments, pCAG-GFP was coelectroporated with pSUPER-mNR1D1#1 together with pCAG-Myc-Nr1d1-R (d) or -Nr1d1-R500H (e). Coronal sections were prepared at P2. Nuclei were stained with DAPI (blue). Dotted lines represent the pial and ventricular surfaces. Bar, 100 μm. **(B)** Quantification of the distribution of GFP-positive neurons in distinct regions of the cerebral cortex for each condition shown in **(A)**. Error bars indicate SD (n = 3); ***p = 0.0005 (bin1; control vs RNAi#1), ***p = 0.0002 (bin1; control vs RNAi#3), ***p = 0.0002 (bin3; control vs RNAi#1), **p = 0.0046 (bin3; control vs RNAi#3), ***p < 0.0001 (bin4; control vs RNAi#1), ***p < 0.0001 (bin4; control vs RNAi#3), *p = 0.0128 (bin5; control vs RNAi#3), ^###^*p* = 0.001 (bin1; RNAi#1 vs RNAi#1 + Nr1d1-R), ^###^*p* < 0.0001 (bin3; RNAi#1 vs RNAi#1 + Nr1d1-R), ^###^*p* < 0.0001 (bin4; RNAi#1 vs RNAi#1 + Nr1d1-R), ^$$$^*p* = 0.0002 (bin1; RNAi#1 + Nr1d1-R vs RNAi#1 + Nr1d1-R500H), ^$$$^*p* = 0.0006 (bin3; RNAi#1 + Nr1d1-R vs RNAi#1 + Nr1d1-R500H), ^$$$^*p* < 0.0001 (bin4; RNAi#1 + Nr1d1-R vs RNAi#1 + Nr1d1-R500H) by Fisher’s LSD. (**C**) Representative images of control and Nr1d1-deficient neurons migrating in lower CP. Bar, 5 μm. (D) pCAG-GFP was coelectroporated with control pSUPER vector, pSUPER-mNR1D1#1 or #3 into cerebral cortices at E14.5. Coronal sections were prepared at P7. Quantification of the distribution of GFP-positive neurons in distinct regions was performed as in (**B**). Error bars indicate SD (n = 3).

To investigate whether functional defects of Nr1d1 induce abnormal brain development which might be related to etiology of ASD, we performed RNAi experiments in embryonic mouse brains. Effects of Nr1d1-silencing on the migration of newly generated cortical neurons were examined with the *in utero* electroporation technique. pSUPER-mNR1D1#1 or #3 was electropolated with pCAG-EGFP into progenitor and stem cells in VZ of E14.5 mice brains. When the localization of transfected cells and their progeny was analyzed at P2, control vector-transfected neurons migrated normally to the superficial layer (bin1; layer II-III) of CP (Fig. 5A a and B). In contrast, cells transfected with pSUPER-mNR1D1#1 or #3 frequently remained in the lower part of CP and the intermediate zone (IZ) (Fig. 5A b and c and B). Given that pSUPER-mNR1D1#3 silences both Nr1d1 and Nr1d2 in COS7 cells, statistically similar phenotypes by pSUPER-mNR1D1#1 and #3 might indicate a minor role of Nr1d2 in cortical neuron positioning. Since pSUPER-mNR1D1#1 was specific for Nr1d1, we used this RNAi vector in the subsequent experiments.

Rescue experiments were then conducted to rule out off-target effects. When pCAG-EGFP was electroporated into the VZ cells with pSUPER-mNR1D1#1 together with pCAG-Myc- Nr1d1-R, the positional defects were significantly rescued at P2 (Fig. 5A d and B). It is notable that the patient-related mutant Nr1d1-R500H could not rescue the phenotype under the same conditions (Fig. 5A e and B). Since cell migration is tightly associated with the cell shape, morphology of Nr1d1-deficient neurons was examined in the lower CP. Consequently, the mispositioned cells frequently showed abnormal phenotypes such as multipolar-like morphology and shapes with a branched leading process (Fig. 5C). When cortical neuron migration was examined at a later time point (P7), migration was still prevented and many Nr1d1-deficient neurons failed to reach their target destination (layers II–III) (Fig. 5D).

Taken together, these results strongly suggest that functional defects of Nr1d1 may disrupt cortical neuron migration and cause abnormal cortical architecture. The mutation c.1499G > A might be implicated in pathogenesis and pathophysiology of ASD.

### Time-lapse imaging of migration of Nr1d1-deficient neurons in cortical slices

**Figure 6:**
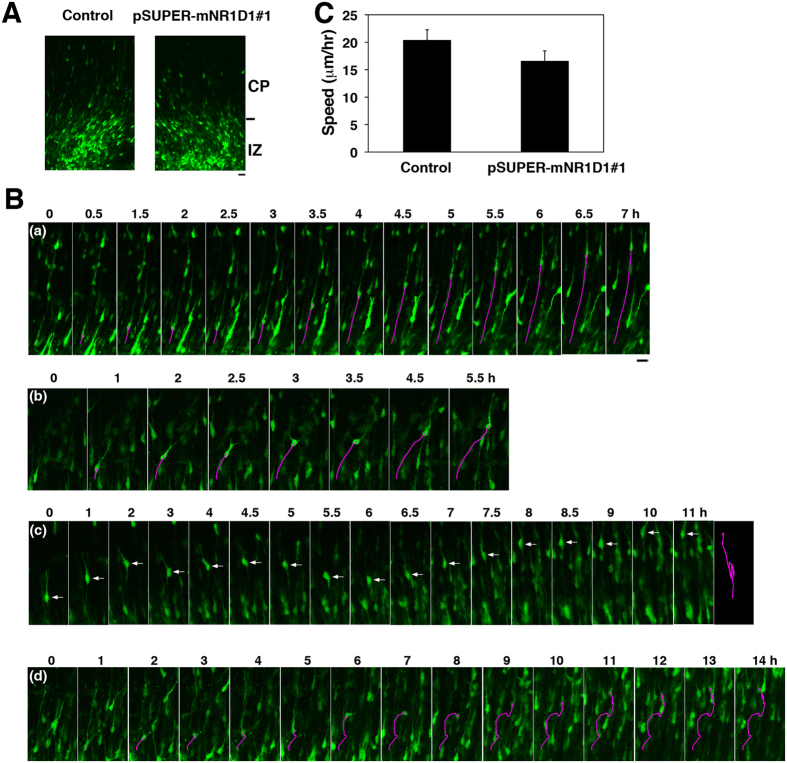
Time-lapse imaging of migration of Nr1d1-deficient neurons. Analyses were repeated 3 times for each case, and the migration pattern was observed for 10 cells in each imaging. Representative results were shown in **(A)** and **(C**). **(A**) Cortical slices at the beginning of slice culture under the confocal microscope were shown. E14.5 cortices were coelectroporated with pCAG-EGFP together with pSUPER control vector or pSUPER-mNR1D1#1, followed by coronal section slice preparation at E16.5 and time-lapse imaging. No difference in transfection efficiency was observed between the experiments. Bars in **(A)** and **(B)**, 20 μm. **(B)** Time-lapse imaging of control **(a)** and Nr1d1-deficient neurons **(b–d)** migrating in CP. **(b)**, **(c)** and **(d)** represent tangential migration, reverse migration and migration in a complex manner, respectively. Migratory tracks of representative cells were traced and shown as red lines **(a,b,d)**. Tracing of the migration of **(c)** was shown at the far right. **(C)** Calculation of migration velocity of control and the deficient neurons in the middle-upper CP. Ten cells were analyzed in each experiment (n = 3). Error bars indicate SD.

The abnormal positioning of Nr1d1-deficient neurons might be caused by reduced migration velocity. Alternatively, the positional defects may be attributable to the migration into aberrant directions in CP rather than the reduced velocity. We thus performed time-lapse imaging of Nr1d1-deficient cortical neurons migrating in IZ and CP. To this end, VZ progenitor and stem cells were electroporated with pCAG-EGFP together with pSUPER-H1.shLuc (control) or pSUPER-mNR1D1#1 at E14.5, and migration was monitored from E16.5. At the beginning of imaging, Nr1d1-deficient cells showed apparently normal phenotypes in upper IZ and the IZ/CP boundary; they were multipolar and some fraction of them were transforming into the bipolar status like control cells (Fig. 6A). Nr1d1-deficient cells appeared to transform normally from multipolar to bipolar at upper IZ as in the case of the control cells. The initiation of radial migration of the deficient cells was also found to be normal after the multipolar-bipolar transition (Supplementary video 1–4). However, at later time points, abnormal morphological phenotypes frequently came to be observed for the deficient cells migrating in CP (Fig. 6B a–d, Supplementary video 1–4). Notably, some Nr1d1-deficient neurons were observed to jump in a tangential direction, under the conditions where control neurons exhibited steady migration in a radial direction (Fig. 6B a and b, Supplementary video 1 and 2). Nr1d1-deficient cells also sometimes displayed sudden upside-down cell polarity change followed by aberrant migration to the reverse direction (Fig. 6B c, Supplementary video 3). In addition, abnormal morphological change in a complex manner was monitored in some deficient neurons during radial migration (Fig. 6C d, Supplementary video 4). While we observed the abovementioned characteristic migration phenotypes frequently, majority of Nr1d1-deficient neurons displayed apparently normal radial migration profile in CP. Thus, their average migration speed was not statistically different from that of control cells (Fig. 6D). The abnormal morphological phenotypes are likely to be observed when the RNAi effect is relatively strong.

### Nr1d1 is not involved in the cell cycle of progenitor and stem cells in VZ

**Figure 7:**
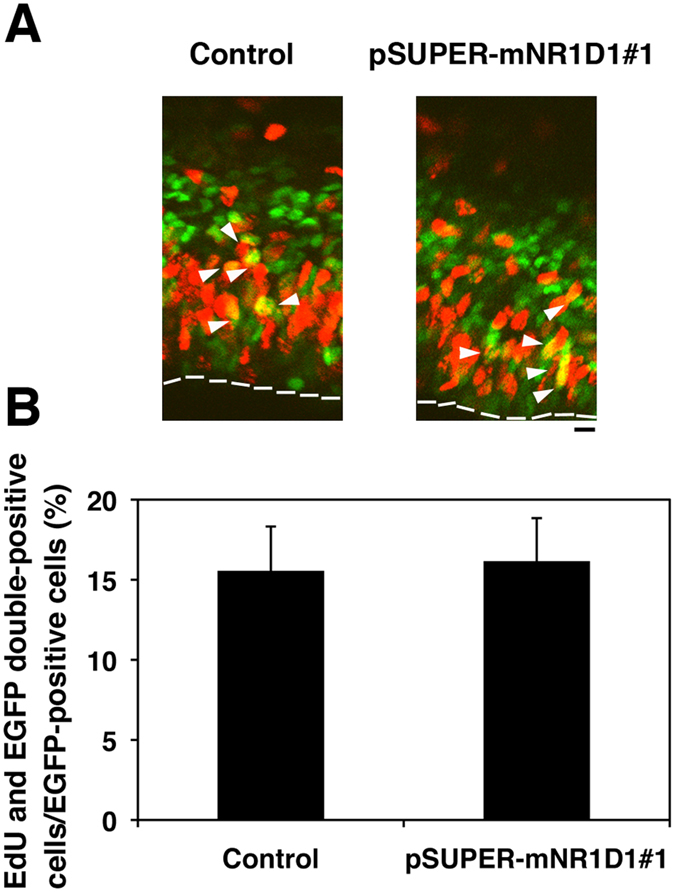
Effects of Nr1d1-silencing on the cell cycle. **(A)** E14.5 cortices were coelectroporated with pCAG-GFP together with control pSUPER vector or pSUPER-mNR1D1#1. The subsequent procedure performed is described in “Materials and methods”. Coronal sections were immunostained for GFP (green) and EdU (red). Dotted lines represent ventricular surface. Bar, 10 μm. **(B)** Quantification of EdU/GFP double-positive cells relative to GFP-positive ones in **(A)**. Error bars indicate SD (n = 3).

Since neuronal migration delay was observed in the case of a prolonged cell cycle^22^, we looked into the possible role of Nr1d1 in the cell cycle regulation of neuronal progenitor and stem cells in VZ. When the impact of Nr1d1-knockdown on the cell cycle was examined by labeling S-phase cells with EdU to detect DNA replication, Nr1d1-deficient cells entered S-phase to a similar extent as the control cells and G1-progression rate not statistically differ between control and deficient cells (Fig. 7A and B). Nr1d1-knockdown was therefore suggested to have little effects on the cell proliferation at VZ/SVZ. Furthermore, the positioning of the EdU/EGFP double-positive cells within VZ/SVZ was not affected by the knockdown (Fig. 7A). Collectively, we conclude that Nr1d1-deficiency does not influence the cell cycle in VZ cells, and that abnormal neuron positioning by Nr1d1-knockdown was attributable to cell migration defects.

### Involvement of Nr1d1 in the interhemispheric axon elongation *in vivo*

**Figure 8:**
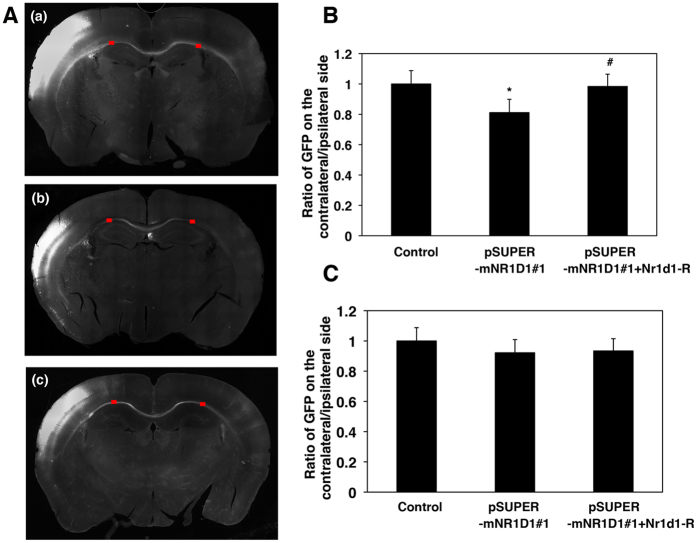
Effects of Nr1d1-knockdown on axon growth *in vivo.* **(A)** pCAG-GFP was coelectroporated with control pSUPER vector (a), pSUPER-mNR1D1#1 (b), or pSUPER-mNR1D1#1 + pCAG-Myc-Nr1d1-R (c) into cerebral cortices at E14.5. Coronal sections were prepared at P2. Bar, 500 μm. **(B)** Estimation of axon growth at P2. GFP signal intensity of callosal axons was measured in the marked areas on the ipsilateral (before entering the corpus callosum (CC)) and contralateral (after leaving the CC) sides, respectively. Then, the ratio of the signal intensity in the ipsilateral area to the contralateral one was estimated. Error bars indicate SD (n = 3); *p < 0.05 (control vs RNAi#1), ^#^*p* < 0.05 (RNAi#1 vs RNAi#1 + Nr1d1-R) by Fisher’s LSD. **(C)** Estimation of axon growth at P10. Quantitative analyses were done as in **(A)**. Error bars indicate SD (n = 3).

Given that ASD clinical phenotypes are associated tightly with impaired synapse functions, functional defects of Nr1d1 may disrupt not only cortical neuron migration but also axon elongation during corticogenesis. We thus analyzed interhemispheric axon projections of Nr1d1-deficient neurons. When Nr1d1 was silenced in the VZ cells at E14.5 and axons were observed at P2, axon density of the deficient neurons became lower after crossing the corpus callosum (CC) when compared to control cells (Fig. 8Aa b and B). It is notable that this phenotype was rescued by Nr1d1-R (Fig. 8Ac and B). Since this phenotype could be due to a developmental delay, we tested if axons of the deficient neurons eventually ended up extending correctly. Consequently, axon growth of the deficient neurons was found to be delayed, but not prevented, since axons extended efficiently into the contralateral cortex at P10 (Fig. 8C). Taken together, Nr1d1 is likely required for axon growth of excitatory neurons from the ipsilateral to the contralateral cortex, and disturbance of which might affect synapse network formation.

### Nr1d1 is involved in the regulation of dendritic arbor formation *in vivo*

**Figure 9:**
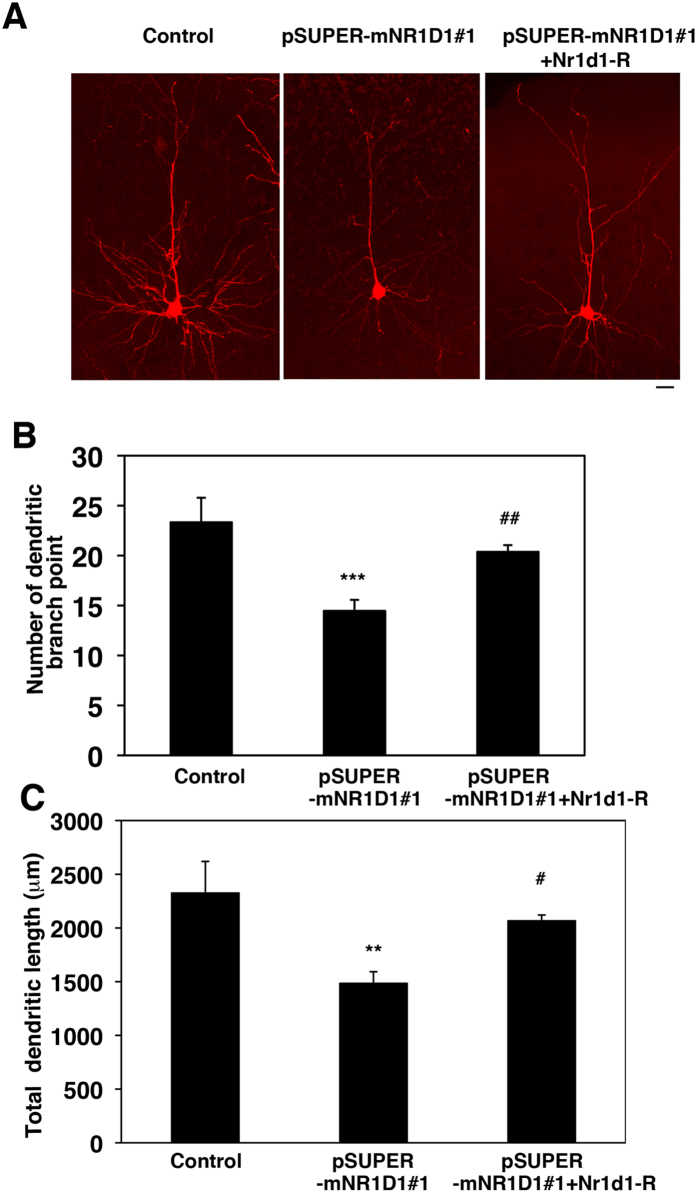
Role of Nr1d1 in the dendrite growth in cortical neurons *in vivo.* **(A)** pCAG-loxP-RFP was electroporated for sparse expression with pCAG-M-Cre together with control pSUPER vector, pSUPER-mNR1D1#1 or pSUPER-mNR1D1#1 + pCAG-Myc-Nr1d1-R into cerebral cortices at E14.5. Analyses were carried out in cortical slices at P30. Representative average Z-stack projection images of RFP fluorescence of cortical neurons in upper CP are shown. Bar, 50 μm. **(B,C)** Number of dendritic branch point **(B)** or total dendritic length **(C)** was calculated on neurons observed in **(A)**. Three brains were analyzed for each experiment; Control, n = 12 neurons; pSUPER-mNR1D1#1, n = 22; pSUPER-mNR1D1#1 + pCAG-Myc-Nr1d1-R, n = 20. Error bars indicate SD; ***p < 0.001 (control vs RNAi#1), *p < 0.01 (control vs RNAi#1), ^##^*p* < 0.01 (RNAi#1 vs RNAi#1 + Nr1d1-R), ^#^*p* < 0.05 (RNAi#1 vs RNAi#1 + Nr1d1-R) by Fisher’s LSD.

We next examined the role of Nr1d1 in dendrite development. Introduction of pSUPER-mNR1D1#1 at E14.5 into VZ cells resulted in highly abrogated dendritic arborization at P7 when compared to the control phenotype (Fig. 9A a and b). Both branch point number and total length of dendrites were lower in the deficient neurons than the matching control cells (Fig. 9B and C). It should be noted that these aberrant phenotypes were rescued by Nr1d1-R (Fig. 9Ac,B and C). Collectively, the obtained data suggest that Nr1d1 plays a crucial role in dendrite growth and maintenance, and that the functional loss of Nr1d1 impairs neuronal connectivity through disturbance of dendrite growth as well as axon elongation. Since the development of the neuronal network is essential for brain function, the clinical features of ASD with *NR1D1* gene abnormalities may reflect the observed cellular phenotypes.

## Discussion

While sleep is thought to be essential for synaptic development and brain maturation^23^, possible interplay of synaptic and clock genes has been proposed in ASD pathogenecity^24^. We have detected mutations in various circadian-related genes, including *NR1D1* by the screening of ASD patients (Fig. 1)^15^. Further screening of ASD patients for *NR1D1* mutations revealed novel mutations, including c.1499G > A (p.R500H), which was predicted to have deleterious effects on gene function based on pathogenic prediction analyses *in silico*. Although the younger brother of the patient was also diagnosed as autistic, no mutation was found in the *NR1D1* gene, suggestive of an abnormality in as yet an unidentified gene(s) when considering numerous causal gene candidates for ASD^25^,^26^. Alternatively, there may also exist a recessive inheritance of a yet unidentified gene in the pathogenesis of the present patient. In such a case, *NR1D1* may be one of many polygenic factors. Further genetic study with a much larger sample size is required to elucidate the pathogenic significance of *NR1D1* gene abnormalities.

We examined the possible contribution of NR1D1 to neuronal development, based on the prediction that the c.1499G > A mutation is pathogenic for this patient as it was located in the ligand-binding region in NR1D1. Since ligand-binding is thought to cause a conformational change in NR1D1 that induces a response and acts as a molecular switch to turn the transcriptional activity on, the c.1499G > A mutation may impair the physiological role of NR1D1 and consequently contribute to the pathogenesis of the patient. In this regard, the mutation was predicted to be “probably damaging” (score 0.946) by PolyPhen-2, “deleterious” (score 0.01) by SIFT and “disease causing” (score 29) by Mutation Transfer analyses.

NR1D1 is known to negatively regulate the expression of circadian clock proteins as mentioned earlier. Meanwhile, Nr1d1 has been reported to participate in neuronal architecture and function during the brain development, through the interaction with Oligophrenin-1, a GTPase-activating protein for Rho small GTPases^20^. Oligophrenin-1 has been demonstrated to regulate dendritic spine morphology^27^, and its gene abnormalities are considered to cause intellectual disability (ID) and cerebellar hypoplasia^28^. Oligophrenin-1 is likely to recruit Nr1d1 to dendrites, reduce its repressor activity and protect Nr1d1 from degradation^20^. Interestingly, while Oligophrenin-1 appears to be involved in the circadian cycle through the expression of clock genes, including Nr1d1, synaptic activity may reciprocally regulate the Nr1d1 accumulation to dendrites and spines^20^. Collectively, functional defects of NR1D1 as well as Oligophrenin-1 are likely to relate to the pathophysiology of neurodevelopmental disorders such as ASD and ID.

Unlike wild type Nr1d1, Nr1d1-R500H could not rescue the migration defects in the knockdown experiments, indicating that functional impairment of Nr1d1 presumably takes part in the etiology of the present ASD case. It is not clear whether c.1499G > A mutation serves as a loss-of-function mutation or dominant negative one. In the latter case, the interaction of the remaining normal NR1D1 with its physiological binding partners might be prevented by NR1D1-R500H under haploinsufficiency conditions. In either case, partial but not complete inhibition of NR1D1 activity may contribute to the abnormal phenotypes observed in this study. In addition to the neuronal migration defects, interhemispheric axon projection and dendritic arbor formation were abrogated when Nr1d1 was silenced in the developing mouse cerebral cortex. Since Nr1d1 is a component of a transcription machinery, inhibition of its function may cause a variety of functional defects in neuronal cells. It remains to be determined if the defects in neuronal network function and/or maintenance really occur in individuals with defective NR1D1 function, and, if they do, how the abnormality determines the clinical features.

Based on the observation in this study, NR1D1 is likely to contribute to cortical development through regulation of neuronal migration and synaptic network formation. The present study suggests that functional defects of NR1D1 may be implicated in the etiology and pathophysiology of ASD and other neurodevelopmental disorders, although further genetic and cell biological analyses are required to fully address the issue.

## Materials and Methods

### Ethics statement

The clinical study was approved by the bioethics committee for human gene analysis at Jichi Medical University (approval number 11–14) in accordance with the principles of the Declaration of Helsinki, and the Ethical Guidelines for Human Genome/Gene Analysis Research by the Ministry of Education, Culture, Science, and Technology, the Ministry of Health, Labor, and Welfare, and the Ministry of Economy, Trade, and Industry of Japan. Written informed consent was obtained from the parents of the patients. We followed the fundamental guidelines for proper conduct of animal experiments and related activity in academic research institution under the jurisdiction of the Ministry of Education, Culture, Sports, Science and Technology, Japan. All the protocols for animal handling and treatment were reviewed and approved by the animal care and use committee of Institute for Developmental Research, Aichi Human Service Center (approval number M-10).

### Samples

We screened Japanese and Caucasian samples of ASD patients for *NR1D1* gene mutation. The patients were diagnosed as autism, Asperger syndrome, pervasive developmental disorder based on the DSM-IV criteria, or ASD based on the DSM-V criteria. DNA samples of 111 Caucasian patients with ASD were obtained from the Autism Genetic Resource Exchange (AGRE) Consortium (Cure Autism Now, Los Angeles, CA). Caucasian control samples from 158 typically developing children were obtained from the Coriell Institute (Camden, NJ). DNA samples from 87 Japanese patients with ASD and 133 Japanese control samples were obtained from the sample stock at the Department of Pediatrics, Jichi Medical University. The intelligence quotient and language ability of the ASD patients varied from severely affected to normal. All lymphocyte samples were obtained from patients with informed consent from themselves or their parents. Lymphocytes were transfected with Epstein-Barr virus to establish lymphoblasts and cultured. Genomic DNA was then extracted using salting-out methods^15^.

### Direct sequencing analysis

Exons in which mutations were detected were verified by Sanger sequencing analyses. PCR was performed to amplify each exon containing a mutation and its neighboring introns. PCR products were analyzed using the 3730xl DNA Analyzer (Applied Biosystems, Foster City, CA). The primer sequence information will be provided on request.

### Genetic data analysis

The sequencing results were aligned to genomic reference sequences and called variants were compared with the reference genome using the 454 integrated software (GS Reference Mapper, Roche). We searched for mutations in the exons of *NR1D1*. A sample was considered mutated if a mutation was present in a minimum of approximately 50% of the confident reads. Genomic reference sequences were ascertained by the NCBI website (http://www.ncbi.nlm.nih.gov/gene/). Each coding polymorphism was compared to the dbSNP (http://www.ncbi.nlm.nih.gov/snp/) and the corresponding reference (rs) number was assigned to previously identified polymorphisms. Japanese mutations were also ascertained by the human database website (http://biosciencedbc.jp/en/) of the National Bioscience Database Center of the Japan Science and Technology Agency. The patients, their siblings and parents were tested for mutations in all exons of *NR1D1*. The hypothesized effects of the mutations on respective protein functions were analyzed using prediction tools PolyPhen-2 (http://genetics.bwh.harvard.edu/pph2/), SIFT (http://sift.jcvi.org/) and Mutation Taster (http://www.mutationtaster.org/).

### Plasmids

Mouse *Nr1d1* and *Nr1d2* cDNAs were obtained from Dr. T. Takumi (RIKEN, Saitama, Japan) and constructed into pCAG-Myc vector (Addgene Inc., Cambridge, MA). pCAG-histone 2B (H2B)-EGFP was used to label chromosomes^29^. pCAG-M-Cre was kindly supplied by Dr. S. Miyagawa (Univ. Osaka, Japan)^30^. pCALNL(loxP-neomycin-loxP)-RFP was made as previously described^29^. For RNAi experiments, pSUPER-RNAi-puro vector (OligoEngine, Seattle, WA) was designed to target three distinct coding sequences in mouse *Nr1d1* cDNA (pSUPER-mNR1D1#1, 5′-GGACCAGACAGTGATGTTC-3′, 1477–1495; pSUPER-mNR1D1#2, 5′-GGCAACACCAAGAATGTTC-3′, 1229–1447; pSUPER-mNR1D1#3, 5′-GTAGAGTTTGCCAAACACA-3′, 1354–1372). Numbers indicate the position from the transcription start site. We used pSUPER-H1.shLuc designed against luciferase as a control RNAi vector^29^. To generate an RNAi-resistant Nr1d1, Nr1d1-R, silent mutations were introduced, as underlined, in the target sequence (5′- GGATCAAACGGTCATGTTC -3′, 1477–1495). The Nr1d1 mutant, Nr1d1-R500H, was prepared using KOD-Plus Mutagenesis kit (Toyobo, Osaka, Japan) with Nr1d1-R as a template. All constructs were verified by DNA sequencing.

### *In situ* hybridization

Coronal sections of mouse brain at embryonic day (E)14, E16 and postnatal (P)0 were probed using a digoxigenin-labeled antisense riboprobe directed against full length mouse *Nr1d1* cDNA as previously described^31^. The sense riboprobe was used for the control experiment.

### Primary antibodies

Polyclonal rabbit anti-GFP and anti-Myc antibodies were generated as described^32^. Polyclonal rabbit antibody against a cytoskeleton-related protein, Sept11, was prepared as described^33^.

### Cell culture, transfection, western blotting and immunofluorescence

COS7 cells were transfected with Lipofectamine 2000 (Life Technologies Japan, Tokyo). SDS-PAGE and western blotting were performed as previously described^34^. Immunofluorescence analyses were carried out as previously described^35^. Alexa Fluor 488- or 568-labeled IgG (Life Technologies Japan) was used as a secondary antibody. Fluorescent images were captured with FV-1000 confocal laser microscope (Olympus, Tokyo, Japan).

### *In utero* electroporation and time-lapse imaging

*In utero* electroporation was carried out essentially as previously described^36^,^37^ at E14.5. At least 5 brains were used for each experiment. Live-imaging analyses were conducted as described previously^31^.

### Quantitative analysis of neuronal migration and axon elongation

Three electroporated brains from different dams were used for each experiment. Distribution of GFP-positive neurons in brain slices was quantified as follows. The coronal sections of cortices containing the labeled cells were classified into 5 bins and IZ as previously described^29^,^38^. The number of labeled cells (more than 100 *per* sample) of at least 5 slices *per* brain was calculated. For estimation of axon growth, GFP signal intensity of callosal axons was measured in the marked areas on the ipsilateral (before entering the CC) and contralateral (after leaving the CC) sides. We then calculated the ratio of the contralateral signal intensity to that in the corresponding ipsilateral side using ImageJ software.

### EdU (5-ethynil-2′-deoxyuridine) incorporation experiment

Cell cycle analysis was performed essentially as previously described^31^. Embryos were electroporated *in utero* with pCAG-H2B-EGFP together with pSUPER-H1.shLuc (control) or pSUPER-mNR1D1#1 at E14.5. GFP and EdU were detected with anti-GFP and Alexa Fluor555 azide (Life Technologies Japan), respectively. The number of cells used for each calculation was more than 100.

### Statistical analysis

Results were expressed as means ± SD. When data were obtained from only 2 groups, Student’s *t*-test was used for comparison. For other experiments, the rate of cell scores was initially analyzed using the one-way analysis of variance (ANOVA). Subsequently, Fisher’s least significant difference test (LSD) was applied to absolute values as a *post hoc* test of multiple comparisons. The level of statistical significance was considered to be *p* < 0.05. Statistical analysis was performed using Statview software (SAS Institute, Cary, NC).

## Additional Information

**How to cite this article**: Goto, M. *et al*. Role of a circadian-relevant gene NR1D1 in brain development: possible involvement in the pathophysiology of autism spectrum disorders. *Sci. Rep.* **7**, 43945; doi: 10.1038/srep43945 (2017).

**Publisher's note:** Springer Nature remains neutral with regard to jurisdictional claims in published maps and institutional affiliations.

## Figures and Tables

**Table 1:** The results of direct sequencing analyses for Autistic spectrum disorders in *NR1D1*.

Base change	Amino acid change	Samples		Inheritance	Control screening	SNP number	PolyPhen-2 analysis	Mutation Taster analysis	SIFT analysis
		Japanese	Caucasian						
c.58A > C	p.S20R	1/87	0/108	Father inherited	0/133	—	Benign	Disease causing	Damaging
c.1012 C > T	p.P338S	0/87	2/111	Mother inherited	0/158	rs143682026	Benign	Polymorphism	Tolerated
c.1031 A > C	p.N344T	0/87	1/111	Mother inherited	0/158	rs145435357	Benign	Disease causing	Tolerated
c.1499G > A	p.R500H	0/87	1/107	*De novo*	0/158	—	Probably damaging	Disease causing	Deleterious

The patient with p.S20R mutation in *NR1D1* has also c.2537 G > A (p.R846Q) mutation in *SHANK2.*
